# Upper respiratory tract disease in European hedgehogs (*Erinaceus europaeus*): Histopathological, microbiological, and clinical aspects

**DOI:** 10.1177/03009858261451485

**Published:** 2026-05-27

**Authors:** Yannick Van de Weyer, Steve Bexton, Joanna Mihr, Mariana C. C. Santos, Flavia Zendri, Valerie Tilston, Dorina Timofte, Guido Rocchigiani, Julian Chantrey

**Affiliations:** 1 Veterinary Anatomy, Physiology and Pathology Department, Leahurst Campus, University of Liverpool, Neston, UK; 2 Royal Society for the Prevention of Cruelty to Animals East Winch Wildlife Centre, King’s Lynn, UK; 3 Royal Society for the Prevention of Cruelty to Animals Mallydams Wood, Hastings, UK; 4 Royal Society for the Prevention of Cruelty to Animals Stapeley Grange Wildlife Centre, Nantwich, UK; 5 Institute of Zoology, Zoological Society of London, Regent’s Park, London, UK

**Keywords:** hedgehog, *Morganella morganii*, nasal cavity, oronasal fistula, *Pasteurella multocida*, rhinitis, trauma

## Abstract

Respiratory disease is a common cause for morbidity and mortality in European hedgehogs (*Erinaceus europaeus*) presented for rehabilitation. Despite frequent suspicions of underlying pulmonary disease, upper respiratory tract disease (URTD) investigations rarely occur. Fifty rehabilitating European hedgehogs that died after exhibiting respiratory clinical signs were necropsied with histological and microbiological investigations of the respiratory tract. Fifty-six percent (n = 28) of these hedgehogs had substantial URTD based on histological assessment of 3 transverse nasal cavity sections. URTD cases presented with moderate to marked lethargy (82%), dyspnea and/or tachypnea (79%), abnormal breathing sounds (75%), nasal discharge (64%), and sneezing (54%). Most hedgehogs with URTD were euthanized, 13 (48%) within 24 hours of admission due to the severity of clinical signs, the others (52%) after a period ranging from 3 to 240 days whilst receiving various treatments. Histologically, substantial URTD cases presented as moderate-to-severe rhinitis (40%), epithelial damage ranging from widespread attenuation to multifocal ulceration (40%) and/or maxilloturbinate atrophy (22%) of the nasal chamber proper, and/or inflammation of the vestibule (20%) or caudal nasal cavity (43%). Rhinitis cases were often bilateral (80%), exudative, and neutrophilic with intraluminal gram-negative bacterial colonies. Bacterial culture yielded *Morganella morganii* (n = 6) and *Pasteurella* spp. (n = 5) from cases with URTD but not from histologically unremarkable noses. Potentially underlying triggers for URTD included trauma, foreign bodies, periodontal disease, oronasal fistula, and nasal fluke. As respiratory clinical signs in hedgehogs are commonly associated with URTD, routine antemortem and postmortem assessments of the nasal and oral cavities are warranted.

The European hedgehog (*Erinaceus europaeus*) is the most frequently presented wild mammalian species at rehabilitation centers in Western Europe. Despite conservation and rehabilitation efforts, populations have been declining in recent years, which resulted in a reclassification to “near threatened” on the International Union for Conservation of Nature Red List.^4,15^ Respiratory diseases are a common cause for morbidity and mortality in rehabilitating European hedgehogs, with trauma and verminous or bacterial pneumonia considered as the main etiologies.^2,3,20,31^ However, literature on alternative causes is sparse, as most studies have focused on pulmonary diseases.^3,30^ Upper respiratory tract disease (URTD) is poorly documented in hedgehogs, despite the crucial role of the olfactory system for food acquisition and communication.^7,29^ Postmortem studies reported suppurative rhinitis in two European hedgehogs,^34^ and one case of an oronasal fistula associated with periodontal disease and systemic *Streptococcus pyogenes* infection.^12^ However, in these studies clinical data were unavailable. The oral and nasal cavities are in close proximity, and oral problems can therefore result in nasal cavity disease.^14^

URTD is relatively well-documented in domestic animals.^6^ Classic examples include atrophic rhinitis in pigs, a polymicrobial disease associated with overcrowding and poor husbandry conditions,^6,16,25^ whereas neoplasia, bacterial, mycotic, allergic, and chronic idiopathic rhinitis are relatively common in companion animals.^6,22,23^ In addition, trauma, foreign bodies, dental disease, nasal fistula, and uremia have also been associated with disease of the nasal cavity in dogs and cats.^6,11,22^ Other than bacterial infections, dysbiosis is thought to play an important role in various URTD.^32^ Nevertheless, our understanding of the differences between healthy and pathological nasal microbiomes, even in companion animals, remains limited. Bacterial populations vary among anatomical regions of the nasal cavity, and determining causal roles for specific isolates is often difficult.^24,32^

Persistent respiratory disease that is nonresponsive to routine treatments presents a major challenge for wildlife veterinarians in hedgehog rehabilitation (personal observation, YVDW). Enabling veterinarians to better understand potential underlying causes would guide diagnostic investigations and can result in quicker decision making, thereby improving animal welfare and utilization of resources within wildlife centers. We hypothesize that URTD is a prevalent underlying problem in hedgehogs that present with respiratory clinical signs nonresponsive to treatment. The aims of this study were to investigate and describe the underlying causes of respiratory clinical signs in European hedgehogs, focusing on diseases affecting the upper respiratory tract; their clinical presentation; associated microbiological isolates; and histopathological lesions.

## Materials and Methods

### Study Population

All hedgehogs from this study were under temporary human care for rehabilitation. Veterinarians working in England with European hedgehogs were recruited via email from 4 Royal Society for the Prevention of Cruelty to Animals wildlife centers, local wildlife rehabilitation centers within 20 miles of Leahurst campus, the University of Liverpool, and 1 wildlife center each in Somerset and Gloucestershire. Inclusion criteria included European hedgehogs that died (euthanasia or natural) with a recent (<48 hours) history of respiratory clinical signs, defined as abnormal breathing sounds, sneezing, coughing, tachypnea, dyspnea, or moderate-to-severe nasal discharge. A small number of animals that died with no history of respiratory clinical signs were included as controls. Euthanasia was a clinical decision based on veterinary discretion and no animals were euthanized for the purpose of this study. Ethical approval was granted by the Institute of Infection Veterinary and Ecological Sciences University of Liverpool ethics committee number VREC1373.

### Sampling Protocol

Targeted postmortem examinations to collect samples from the cardiorespiratory tract were performed by veterinarians from wildlife centers, or by the lead investigator (YVdW) when these centers were located within a 20-mile radius from Leahurst campus. A document with the postmortem protocol and sampling instructions was provided to participating veterinarians. Key points included macroscopic examination for any signs of trauma, dental disease, assessment of diaphragmatic integrity, collection of a nasal swab, and removal of the cardiorespiratory pluck and head for preservation in neutral-buffered formalin. All postmortem examinations were conducted less than 48 hours after death, with preservation of the carcass in a refrigerator.

### Histology

Standardized, 5 mm thick transverse sections were obtained from the nasal cavity after ≥48 hours of fixation in 10% neutral-buffered formalin. The main section used for interpretation of the nasal cavity in all animals was obtained by cross-sectioning the maxilla with a diamond saw from the nasal planum to the first premolar based on “section 2” as previously described.^29^ Another section to assess the vestibule and skin was obtained from the distal snout when available.^29^ A third, more caudal nasal cavity section was taken for all controls and a subset (n = *40*) of diseased animals. Histology was also performed on the trachea, lungs, pulmonary lymph node, and heart for all hedgehogs.

Nasal sections were placed in cassettes and underwent slow decalcification in formic acid (RDF, Cellpath) for 3-4 days at room temperature prior to embedding. Sections (4–5 µm thick) of paraffin-embedded tissues were cut on a microtome, transferred onto glass slides, and stained with hematoxylin and eosin for examination by light microscopy. Gram stains were conducted for all cases with visible bacterial colonies at 400× magnification. Giemsa, Grocott methenamine silver, and Ziehl-Neelsen acid fast stains and periodic acid-Schiff reactions were performed on a subset of animals based on hematoxylin and eosin and microbiological findings.

Full histological assessment was conducted for the nasal chamber proper hematoxylin and eosin section, which led to the categorization of 11 histological features, including turbinate atrophy, rhinitis, epithelial damage, periodontal disease, nasal trauma, nasal septum deviation, luminal bacterial colonies, foreign bodies, epithelial hyperplasia or metaplasia, active bone resorption, and vomeronasal organ involvement (Supplemental Table S1). Epithelial attenuation or hyperplasia and osteoclast numbers were determined based on deviation from reference ranges described in healthy hedgehogs.^29^ Other nasal cavity sections were mainly assessed for inflammatory changes and the presence of pathogens and/or foreign bodies. Lower respiratory tract organs were only assessed for inflammatory changes.

### Bacterial Culture

Nasal transport swabs (eSwabs, COPAN Group, Murrieta, California, US) were collected during postmortem examination according to a standardized protocol. Initially, nasal swabs were rarely taken because bacterial URTD was not suspected and veterinarians struggled to access the nasal cavity with standard e-swabs provided, a problem which was solved by distributing the smallest possible size and encouraging routine inclusion. One nasal cavity was swabbed per individual, prioritizing a nostril with discharge when applicable. Samples were kept refrigerated and submitted as soon as possible after collection, and always within 7 days. Specimens were processed at the University of Liverpool Veterinary Microbiology Diagnostic Laboratory for bacterial culture at 37°C for 5 days under aerobic conditions using nonselective 5% sheep blood agar (Oxoid, Basingstoke, UK). In addition, selective media were used for the specific isolation of fastidious organisms, for example, chocolate horse blood agar incubated under capnophilic atmosphere. Finally, samples were enriched in Rappaport-Vassiliadis broth and subsequently cultured on xylose lysine deoxycholate and brilliant green agar plates for the selective isolation of *Salmonella* spp. (all selective media from Oxoid, Basingstoke, UK). Organisms were identified by matrix-assisted laser desorption/ionization time-of-flight mass spectrometry (MALDI-TOF MS) (Bruker Daltonics, Bremen, Germany) with a score >2. Antibiotic susceptibility testing was performed by disk diffusion for organisms with likely clinical significance to respiratory disease (eg, *Bordetella bronchiseptica*, *Pasteurella multocida*, *Histophilus somni*, *Salmonella* spp., *Pseudomonas aeruginosa*, *Escherichia coli*, *Staphylococcus aureus*, and *Streptococcus pyogenes*) and for other organisms isolated in pure or predominant growths. Disk diffusion methodology and interpretation were carried out according to Clinical and Laboratory Standards Institute guidelines using small animal clinical breakpoints.^8,9^ The antibiotic susceptibility testing panel included parenteral antibiotics commonly used to treat European hedgehogs: amoxicillin-clavulanic acid (20-10 µg), trimethoprim-sulfamethoxazole (1.25-23.75 µg), enrofloxacin (5 µg), marbofloxacin (5 µg), oxytetracycline (30 µg), clindamycin (2 µg), and gentamycin (10 µg). Upon detection of *Salmonella* spp., isolates were referred to the Animal and Plant Health Agency for further typing and antibiotic susceptibility testing.

### Clinical Signs

A brief questionnaire enquiring about the signalment and clinical history of each animal was provided and requested along with the samples.

### Statistics

For statistical analyses, hedgehogs were separated into 2 groups based on histological severity of the URTD. The first group, considered as “convincing URTD cases,” was defined as moderate-to-severe rhinitis on any nasal cavity section and/or moderate-to-severe epithelial damage (erosion/ulceration) and/or turbinate atrophy on the main nasal chamber (first premolar) section. The second group comprised animals with no or mild rhinitis and/or turbinate atrophy. The most prevalent bacterial isolates were compared between groups using Fisher’s exact test in R (R-4.1.0, RStudio, Boston, MA, USA). Descriptive statistics were provided for histological features and for clinical signs observed in convincing URTD cases.

## Results

### Study Population

A total of 50 hedgehogs with clinical respiratory disease, and 5 controls were sampled for the study between November 19, 2023, and December 18, 2024. Out of these 50 animals, 29 were males, 20 females, and for 1 of undetermined sex. Thirty-eight were considered adults and 12 juveniles (<1 year of age) by the submitting wildlife veterinarians based on a combination of size, weight, and season of admission.^2^ Most animals (nearly 50%) came from Norfolk and adjacent counties, followed by Cheshire and East Sussex.

### Histological Findings

Based on histological evaluation of the first premolar section, 72% (36/50) of the animals had rhinitis. This was bilateral for 81% (29/36) of the animals. Neutrophils were the predominant leukocyte type for 92% (33/36) of the animals and were intraepithelial, intra- and perivascular, and frequently intraluminal as part of an exudate. The remaining 3 cases had mild to moderate rhinitis characterized by mucosal lymphoplasmacytic infiltrates with occasional Mott cells. Turbinate atrophy was present in 38% (19/50) of the hedgehogs and was graded mild for 8, moderate for 3, and severe for 8 animals (Fig. 1a). Active bone resorption (Fig. 1b) was observed in 30% (15/50) of the hedgehogs. Epithelial damage was observed in 70% (35/50) of the animals and was considered mild (attenuation) for 15, moderate (>25% attenuation/erosion, Fig. 1c) for 5, and severe (>50% attenuation/multifocal ulceration, Fig. 1d) for 15 hedgehogs. Forty percent (20/50) of the hedgehogs had periodontal disease, 2 with an oronasal fistula and another 2 had a tooth root abscess with concurrent focal maxillary osteomyelitis and bone lysis (Fig. 2). Trauma to the nasal cavity was suspected histologically for 8 animals based on a fractured maxilla (Fig. 2c) with occasional mucosal edema, hemorrhage, and thrombosis in 6 animals, whereas 2 other animals had marked subcutaneous and mucosal hemorrhage. A foreign body compatible with plant material was observed within the nasal chamber proper lumen of 3 hedgehogs from this study (Fig. 2d). For one, this resembled a grass seed (Fig. 2e). Two foreign bodies were surrounded by exudate with bacterial colonies (Fig. 2f) and associated with severe bilateral pyogranulomatous rhinitis. The third had only mild, acute unilateral rhinitis. The prevalence of other histological features of the nasal chamber proper is provided in Table 1.

**Figure 1.**
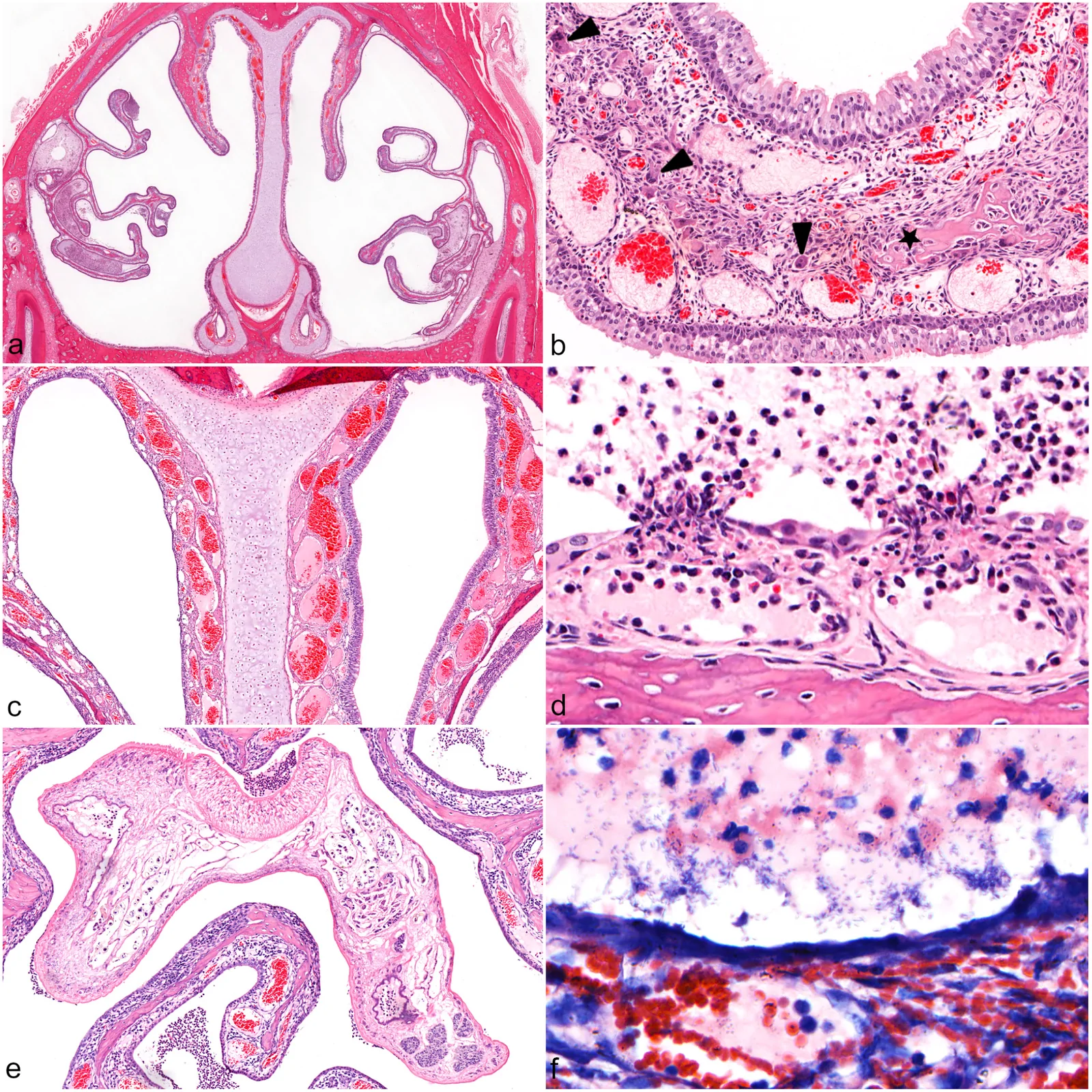
Histology of nasal chamber proper lesions in European hedgehogs with a history of respiratory disease. (a) Rhinitis with severe maxilloturbinate atrophy. Hematoxylin and eosin (HE). (b) Rhinitis. Marked bone lysis (star) with resorption of the maxilloturbinate lamellae. There are numerous osteoclasts (arrowheads) around the bone and numerous leukocytes within the epithelium. HE. (c) Marked unilateral (left in image) attenuation of the dorsal medial meatus epithelium consistent with moderate epithelial damage. HE. (d) Severe acute rhinitis and epithelial damage. Neutrophils are recruited from the mucosal blood vessels and migrate into the nasal lumen through multifocal crater-like ulcerations in the epithelium. Bacteriology yielded *Aeromonas eucrenophila* and *Raoultella terrigena*. HE. (e) Trematode within the nasal chamber proper associated with mild rhinitis. HE. (f) Rhinitis. Attenuated nasal mucosal epithelium with mostly individual coccobacillary bacteria at the apical surface. Bacteriology yielded pure growths of *Morganella morganii*. Giemsa.

**Figure 2.**
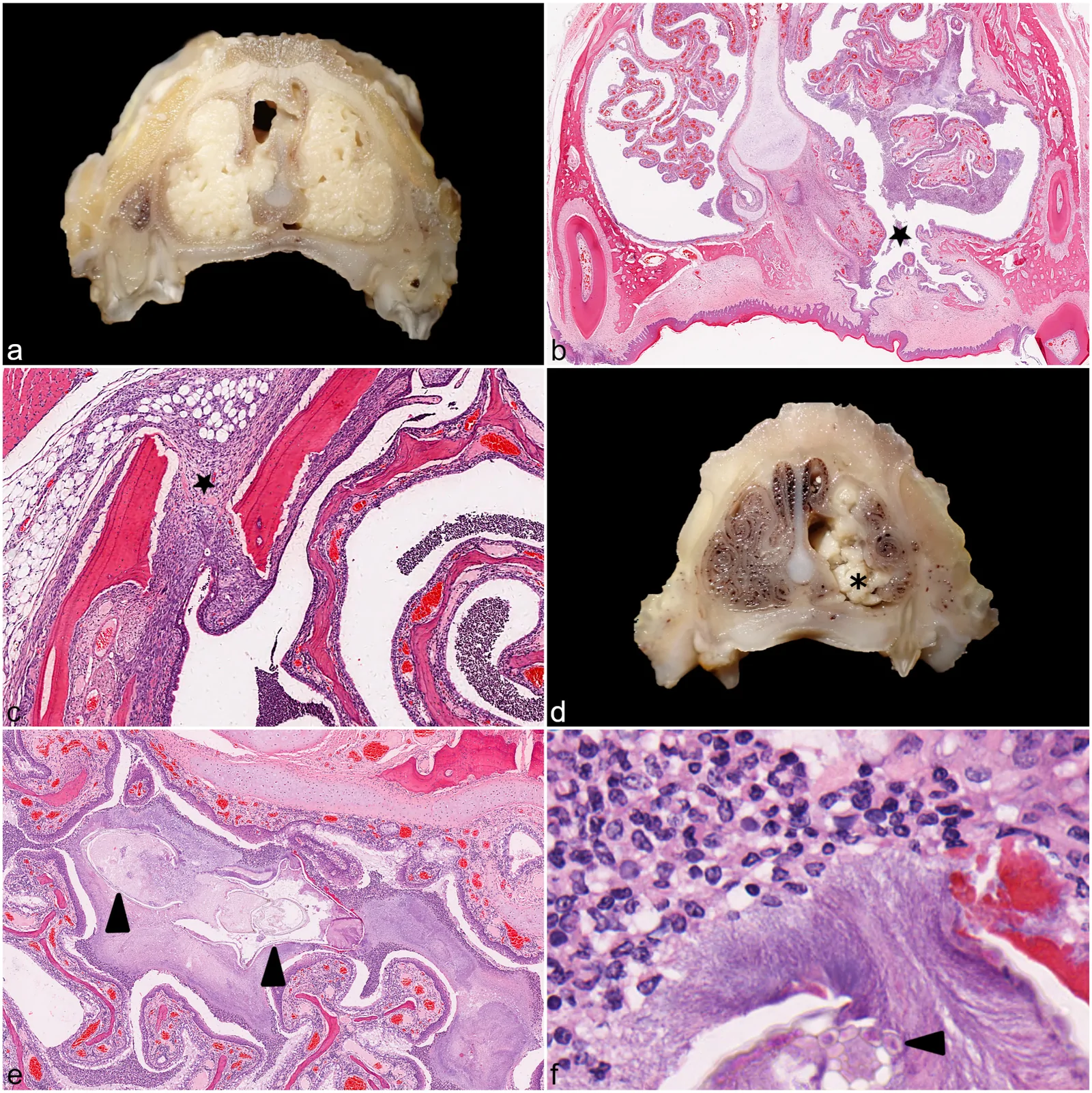
Gross and histological features of nasal inflammation associated with periodontal disease, trauma, or foreign body material in European hedgehogs. (a) Severe, bilateral suppurative rhinitis. Both nasal cavities are occluded by thick yellow exudate with atrophy of the maxilloturbinates and septum deviation with effacement. This animal had severe periodontal disease with isolation of *Morganella morganii* and *Escherichia coli* from the nose. Formalin-fixed specimen. (b) Unilateral rhinitis associated with ipsilateral oronasal fistula (star). Hematoxylin and eosin (HE). (c) Trauma-associated rhinitis. Maxillary fracture (star) with hypercellular nasal mucosa and intraluminal exudate. HE. (d) Severe unilateral rhinitis. Maxilloturbinates of the right nasal cavity are severely atrophied and replaced by thick yellow exudate (pus - asterisk). The contralateral nasal mucosa is edematous. This animal had a nasal foreign body and bacteriology yielded *Escherichia coli* and *Proteus penneri*. Formalin-fixed specimen. (e) Histology of unilateral rhinitis in (d). The left nasal lumen contains a grass seed (arrowheads) surrounded by exudate. HE. (f) Higher magnification of (e) showing plant material (arrowhead) surrounded by bacteria, macrophages, and neutrophils. HE.

**Table 1.** Histological nasal features of 50 European hedgehogs with clinical upper respiratory tract disease.

Histological feature	Absent (n)	Mild (n)	Moderate (n)	Severe (n)
Turbinate atrophy	31	8	3	8
Rhinitis	14	16	6	14
Epithelial damage	15	15	5	15
Periodontal disease	30	11	4	5
Histological feature	Absent (n)		Present (n)	
Nasal trauma	42		8	
Septum deviation	40		10	
Intraluminal bacterial colonies	29		21	
Foreign bodies	47		3	
Epithelial hyper- or metaplasia	36		14	
Active bone resorption	35		15	
Vomeronasal organ involvement	42		8	

Gram stain was performed on tissues from 22 animals, periodic acid-Schiff reaction was performed on tissues from 12 animals, Giemsa stain was performed on tissues from 8 animals, and Ziehl-Neelsen acid fast stain was performed on tissues from 6 animals. Only Gram and Giemsa stains (Fig. 1f) were contributory in highlighting bacteria. Small numbers of intraluminal periodic acid-Schiff-positive fungal spores were observed for 2 animals but given the absence of epithelial invasion or damage, they were considered incidental/postmortem. The nasal chamber proper from 1 hedgehog contained an acoelomate parasite with a prominent sucker; an intestinal tract with ingested exudate; and a reproductive tract with numerous ovoid, 25-µm-long, yellow, operculated ova, compatible with an adult trematode (Fig. 1e). Two other hedgehogs had similar ova within the nasal lumen, but no adult parasites were found. All 3 cases with intranasal fluke stages had mild (n = 2) to severe (n = 1) rhinitis in the absence of trauma, foreign bodies, or substantial dental disease.

One hedgehog with severe lytic necrosis of the nasal mucosa had concurrent necrosuppurative bronchopneumonia associated with *B. bronchiseptica*, but the same bacterium could not be isolated from the nasal swab. Several animals had goblet cell hyperplasia of the dorsomedial meatus, but because the epithelium in this area was often necrotic/ulcerated in severe rhinitis cases, this was not included as a feature for assessment. Multiple animals had dilation of mucosal blood vessels or edema, but this proved difficult to quantify and was therefore also not routinely included in the assessment.

Vestibule sections were available for 45 hedgehogs and the caudal section was available for 40 diseased hedgehogs. Thirty-eight percent (17/45) of hedgehogs had inflammation of the nasal planum and vestibule, some with partial to near complete occlusion of the nares (Fig. 3a, c). Inflammation was considered mild for 8 animals, moderate for 4, and severe for 5. Six of 9 moderate-to-severe cases had no significant changes to the caudal parts of the nasal cavity. All but 1 of these inflammatory processes were characterized by numerous neutrophils, fibrin, and cellular debris, and several had granulation tissue. Occasional nasal planum depigmentation associated with pigmentary incontinence and dermal fibrosis was noted, as well as cartilage fragmentation and mostly superficial, gram-negative bacterial colonies and/or plant material. Sixty-three percent (25/40) of hedgehogs had inflammation of the caudal nasal cavity. This was mild for 8, moderate for 4, and severe for 13 hedgehogs. The mucosal inflammation type was typically more chronic in the caudal nasal cavity compared to the rostral sections, with approximately half of cases having substantial lamina proprial aggregates of plasma cells, fewer lymphocytes, and occasional Mott cells. Discrete mucosal lymphoid aggregates and follicular structures were noted for 5 cases. Neutrophils were mostly observed in the lumen as part of an exudate but were also intraepithelial. Five animals had variable atrophy of the glandular structures. One caudal rhinitis case was associated with a bacterial pseudomycetoma (Fig. 3d), likely originating from the skin and resulting in severe dermatitis, panniculitis, osteomyelitis with lysis of the maxilla, glandular atrophy, and mucus entrapment within the frontal recess. Histology revealed severe pyogranulomatous inflammation with numerous multinucleated giant cells and Splendore-Hoeppli material surrounding very small elements, presumably bacterial colonies. Gram, Ziehl-Neelsen, and Grocott stains and periodic acid-Schiff reactions were noncontributory to identifying the bacteria, and a nasal swab was unavailable.

**Figure 3.**
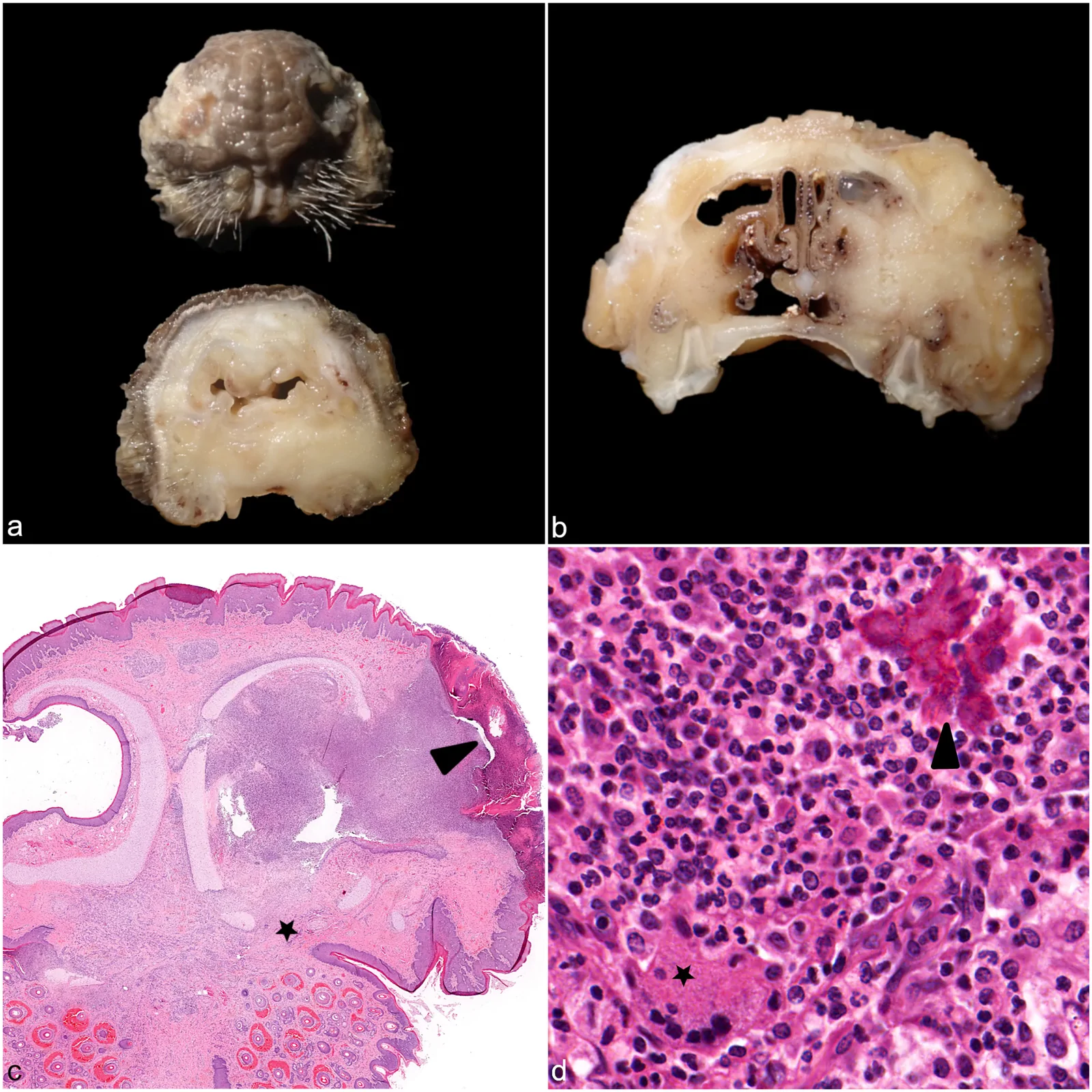
Gross and histological features of nasal bacterial pseudomycetoma. (a) Rostral nasal planum (top) and vestibule (bottom). There is severe unilateral stenosis of the right nostril with granulation tissue narrowing the vestibule. Formalin-fixed specimen. (b) Deep nasal cavity with bacterial pseudomycetoma-associated rhinitis, sinusitis, osteomyelitis, and dermatitis. Formalin-fixed specimen. (c) Histology of the rostral nasal cavity shown in (a). There is a serocellular crust (arrowhead), severe neutrophilic inflammation with deeper granulation tissue, and cartilage fragmentation (star). Hematoxylin and eosin (HE). (d) Histology of the deep nasal mucosa in (b). There is marked pyogranulomatous inflammation with multinucleated giant cells (star) and Splendore-Hoeppli material (arrowhead). HE.

Of the 28 animals with convincing URTD, 39% (n = 11) had moderate-to-severe tracheitis and/or pneumonia. A detailed description of the pathological features of the lower respiratory tract and other organs is outside the scope of this study.

### Controls

Nasal cavities of control animals were mostly unremarkable, with mild lymphoplasmacytic rhinitis noted in 1 hedgehog and mild neutrophilic rhinitis, mild epithelial attenuation, and goblet cell hyperplasia in another. Rostral and caudal nasal cavities from controls were unremarkable with the exception of lymphoid hyperplasia of the caudal nasal cavity in 1 control.

### Microbiological Findings

Nasal swabs from 30 animals were received. Sixty-one bacterial isolates from 24 different genera were obtained. The most common bacterial isolates were *Escherichia coli* (9/30, 30%); *Proteus* spp. (8/30, 27%); *Morganella morganii* (6/30, 20%); *Mammaliicoccus sciuri (6/30, 20%); Pasteurella spp. (5/30; 17%), including P. multocida, P. canis*, and *P. oralis; Aeromonas* spp. *(3/30, 10%), Staphylococcus* spp. *(3/30, 10%), Lactococcus garvieae* (3/30, 10%), and *Enterococcus faecalis* (3/30, 10%). In addition, there were several single species isolates including 1 case of *Salmonella enteritidis* serogroup D1, sequence type 183, phage type 11. The latter was also isolated from the lungs of the same animal. A comparison of the most prevalent isolates between substantial URTD cases and cases with histologically insignificant URTD is provided in Table 2.

**Table 2.** Nasal bacterial isolates from 30 European hedgehogs.

Group	*Escherichia coli*	*Proteus* spp.	*Morganella morganii*	*Mammaliicoccus sciuri*	*Pasteurella* spp.
No-to-mild URTD (n = 11)	2	2	0	3	0
Moderate-to-severe URTD (n = 19)	7	6	6	3	5
*P*-value^a^	.42	.67	.06	.64	.13

a *P*-value of bacterial isolate and moderate-to-severe URTD.

Abbreviation: URTD, upper respiratory tract disease.

There was a trend (*P* = .06) between URTD and *M. Morganii*, but this did not reach statistical significance at a level of .05.

Antibiogram results suggested that *Pasteurella* spp. were often susceptible to all tested antibiotics, whereas nearly all isolates from the nasal cavity were susceptible to gentamycin (Supplemental Table S2).

### Clinical Signs

Cases with convincing URTD presented with moderate to marked lethargy (23/28), dyspnea and/or tachypnea (22/28), abnormal breathing sounds (21/28), nasal discharge (18/28), and sneezing (15/28). Abnormal breathing sounds were further specified as stertor (9/21), stridor (6/21), and/or wheezing (9/21). Nasal discharge was often mucopurulent (11/18), serous (5/18), and/or hemorrhagic (5/18). Most (27/28) URTD hedgehogs were euthanized, 13 within 24 hours of admission due to the severity of the clinical signs, which often included open mouth breathing and/or severe lethargy. The others (14/27) were euthanized after a period ranging from 3-240 days (median = 7 days) whilst receiving various treatments including courses of antibiotics and anthelmintics. From the latter group, 10 failed to gain or lost weight, and 6 had bouts of anorexia.

## Discussion

Fifty-six percent of hedgehogs with clinical respiratory disease had histologically convincing URTD in this study; of these, 61% did not have other relevant changes in the lower airways. Such findings suggest that URTD is highly prevalent in rehabilitating European hedgehogs and that it can be associated with clinical signs. Common clinical signs (as per the inclusion criteria) included dyspnea, tachypnea, abnormal respiratory sounds, mucopurulent to hemorrhagic nasal discharge, and sneezing, comparable with URTD in companion animals.^6,11^ Moreover, the prevalence of nasal discharge and sneezing may be underestimated because several animals were euthanized shortly after admission.

The nasal planum and vestibule had moderate-to-severe acute-to-subacute inflammation with partial occlusion of the nares by inflamed granulation tissue in 9 hedgehogs. This was considered the main reason for respiratory distress in 6 animals, given that their caudal nasal cavity and lower respiratory tract sections were histologically unremarkable. Inflammation and granulation tissue often followed a linear pattern moving inwards from dorsolateral and with partial fragmentation of the cartilage. These findings suggest that trauma, whether from an animal bite, strimmer, or other origin, may be the most plausible cause for these rostral nasal lesions.^2,10^ Intralesional gram-negative bacterial colonies were occasionally observed, but these were considered opportunistic infections.

Maxillary fractures were relatively common in hedgehogs from this study, and it is plausible that subsequent breach in structural integrity of the nasal mucosa resulted in significant URTD. Three of these animals had infected nasal skin wounds and isolation of *Pasteurella canis* from the nose of 2 may imply a predator attack such as a dog, cat, fox, or badger.^1^ Two hedgehogs with maxillary fractures did not meet our criteria for convincing URTD, likely because animals were euthanized shortly after trauma occurred with no time to mount an inflammatory response.

Two out of 3 hedgehogs with intranasal plant material had severe rhinitis, in the absence of trauma or significant periodontal disease, suggesting that inhalation of plant material can trigger URTD just like in companion animals.^11,22^ Although frequently associated with unilateral discharge and rhinitis in dogs and cats,^11^ both hedgehogs with severe foreign body-associated URTD had bilateral inflammation, with one presenting with bilateral mucopurulent discharge and the other with severe dyspnea and lethargy. This observation may represent a later stage of inflammation, which wild hedgehogs often present with for veterinary care.

Oral cavity lesions, especially those affecting the palate, maxilla, or pharynx, can give rise to nasal cavity pathology.^14^ Two URTD hedgehogs from this study had dental abscesses and another two had oronasal fistulas with focal resorption of the maxillary bone (Fig. 2b). Oronasal fistulas can be congenital, but they usually develop secondary to periodontal disease or trauma in companion animals.^6,12,28^ Chronic inflammation follows persistent migration of oral bacteria into the nasal cavity. These findings emphasize the need for thorough oral cavity examinations in hedgehogs with respiratory clinical signs, especially for adults with periodontal disease.

Atrophic rhinitis in pigs is a polymicrobial disease that requires a combination of viral and bacterial agents, typically including *Pasteurella multocida* and *B. bronchiseptica* combined with predisposing factors.^16,25^ Our findings suggest a similar scenario for European hedgehogs because there was no single unifying or dominant bacterial species, and underlying conditions such as trauma or foreign bodies were common. In this study, *Pasteurella* spp. were isolated from the nasal cavities of five hedgehogs, all of which had convincing URTD. Three cases involved *P. multocida*, two of which had head trauma presumably associated with a predator attack. Although not isolated from any of the nasal cavities in this study, one hedgehog had severe necrosuppurative pneumonia associated with *B. bronchiseptica*. Based on established pathogenicity in domestic animals,^6,27^ it is plausible that this bacterium also contributed to the development of the severe necrotizing URTD in this animal. Three out of six hedgehogs with *M. morganii* had severe maxilloturbinate atrophy, and another two had increased osteoclast activity. In human medicine, *M. morganii* is considered an opportunistic pathogen of interest because of increased levels of resistance and virulence, with reported cases of osteomyelitis in humans and reptiles.^17,21,33^ Therefore, *M. morganii* may be associated with an environment that favors bone resorption in hedgehogs, like *B. bronchiseptica* in pigs. However, experimental studies are warranted to further investigate this hypothesis. It is worth noting that bacteria were rarely observed within the lamina propria of affected hedgehogs. The same can apply to porcine atrophic rhinitis cases, whereby bacterial toxins, rather than direct tissue damage, are considered the main driver in bone resorption.^16,25^

*S. enteritidis* sequence type 183 phage type 11 is the most isolated strain from European hedgehogs in Great Britain.^19^ The positive animal from this study had systemic salmonellosis, and despite severe fibrinosuppurative inflammation of the nares and vestibule, there were no obvious lesions in more caudal nasal cavity sections. Trauma to the nasal planum was considered the most likely cause of the snout dermatitis, and there was no evidence of a ventral proliferative rhinitis as reported with *S. enterica* subsp. *diarizonae* in sheep.^18^ Therefore, salmonellosis is not currently considered a major contributor to URTD in European hedgehogs.

To our knowledge, trematodes have not previously been described within the nasal cavity of European hedgehogs,^26^ and it is difficult to conclusively identify them based on histomorphology alone. *Brachylaemus erinacei* is a relatively prevalent fluke in European hedgehogs in Britain with matching ova size and morphology to our case, but according to limited literature, its predilection sites are the intestine and bile ducts.^13,26^ Despite being associated with mild neutrophilic to mixed rhinitis and seemingly attached to the nasal epithelium with its sucker in this case, more research is needed to obtain conclusive speciation and to establish whether the nasal cavity is an incidental or intended location for this parasite’s life cycle.

The observation that chronic inflammatory infiltrates were more common in caudal nasal cavity sections compared to rostral ones, could suggest that, at least for some animals, the URTD processes started here. This may, for example, be the case for ascending bacterial infections from the nasopharynx. Alternatively, it could simply reflect chronic irritation unrelated to the acute changes taking place in the nasal chamber proper and vestibule. Moreover, it should be noted that caudal nasal cavity sections naturally contain mucosal associated lymphoid tissues, which may facilitate a local mononuclear response.

The results of this study need to be interpreted in light of several limitations. Firstly, a single nostril was sampled per animal for bacteriology. Although clinically affected nostrils were chosen where applicable, and URTD was often bilateral, nasal microflora may differ between the right and left nasal cavities. Moreover, contamination is common for this location and establishing causality of isolated bacterial agents challenging. Thus, isolates obtained from nostril swabs may not necessarily reflect the nasal microflora, or the bacterial population associated with disease of the caudal nasal cavity.^24,32^ Hence, although pure/heavy growths of *Pasteurella* spp. or *M. morganii* combined with respiratory clinical signs may be suggestive of URTD, a larger sample size is recommended to further characterize the healthy versus pathogenic nasal microflora in European hedgehogs. Maxillary trauma and foreign bodies are highly plausible causes for the observed URTD, but establishing causation often remained speculative for dental disease except for cases with evidence of dental fistula or tooth root abscesses. Additional nasal cavity sections may help to increase the odds of finding a foreign body or oronasal fistula, but this was outside of the budget for this study. More thorough examination of the oral cavity and medical imaging may also improve diagnosis.^14^ Nearly 40% of hedgehogs with convincing URTD had concurrent convincing lower respiratory tract pathology. It is therefore difficult to determine which, if not a combination of both, was responsible for the reported clinical signs for those cases. Further studies investigating lower respiratory tract diseases, and ways to differentiate these from URTD antemortem, are warranted.

Finally, because of financial and participant’s time limitations, only organs from the cardiorespiratory tract were routinely analyzed. Diseases in other organ systems can uncommonly result in respiratory clinical signs and histological alterations of the respiratory tract.^5,6,22^ For example, uremia has been associated with nasal cavity ulceration in dogs,^22^ but renal histology and biochemistry results were often unavailable for hedgehogs in this study. As such, these cases could have been missed.

## Conclusions

URTD appears common in this European hedgehog population, and it should be considered as an important differential diagnosis for cases presenting with respiratory clinical signs. URTD is often histologically characterized by an exudative, necrotizing, neutrophilic rhinitis with gram-negative bacterial colonies, and it may progress to marked turbinate atrophy. Potential underlying triggers include trauma to the maxilla or rostral nasal planum, periodontal disease, inhaled plant material, nasal flukes, and oronasal fistula. Antemortem investigations of animals that are presented with significant respiratory clinical signs should include radiographs and thorough oral cavity examinations. A comprehensive hedgehog postmortem examination should include routine macroscopic and ideally microscopic assessment of the nasal cavity.

## Supplemental Material

sj-pdf-1-vet-10.1177_03009858261451485 – Supplemental material for Upper respiratory tract disease in European hedgehogs (Erinaceus europaeus): Histopathological, microbiological, and clinical aspects Supplemental material, sj-pdf-1-vet-10.1177_03009858261451485 for Upper respiratory tract disease in European hedgehogs (Erinaceus europaeus): Histopathological, microbiological, and clinical aspects by Yannick Van de Weyer, Steve Bexton, Joanna Mihr, Mariana C. C. Santos, Flavia Zendri, Valerie Tilston, Dorina Timofte, Guido Rocchigiani and Julian Chantrey in Veterinary Pathology
