# Supplementary material for: Upper respiratory tract disease in European hedgehogs (Erinaceus europaeus): Histopathological, microbiological, and clinical aspects
Source: Vet Pathol. 2026 May 27;63(6):1144–54. doi: 10.1177/03009858261451485 (PMC13714209; doi:10.1177/03009858261451485)
Supplement: Supplementary file 1 [file sj-pdf-1-vet-10.1177_03009858261451485.pdf]

Supplemental Materials

Upper respiratory tract disease in European hedgehogs (*Erinaceus europaeus*): Histopathological, microbiological and clinical aspects

Yannick Van de Weyer, Steve Bexton, Joanna Mihr, Mariana Santos, Flavia Zendri, Valerie Tilston, Dorina Timofte, Guido Rocchigiani, Julian Chantrey

Supplemental Table S1. Histological features used to assess the nasal chamber proper and adjacent structures on a single transverse section taken at the 1st premolar in European hedgehogs with clinical respiratory disease.

Histological Feature	Absent	Mild	Moderate	Severe
**Turbinate atrophy**	Normal maxilloturbinate size	Maxilloturbinates are reduced by 10-25%	Maxilloturbinates are reduced by 25-50%	Maxilloturbinates are reduced by >50%
**Rhinitis[a]**	Only resident leukocytes are present	10-25% of nasal mucosa contains inflammatory aggregates	25-50% of nasal mucosa contains inflammatory aggregates	>50% of nasal mucosa contains inflammatory aggregates
**Epithelial damage**	The nasal epithelium is mostly unremarkable with <10% affected by erosion (no ulcer)	10-25% of nasal epithelium is attenuated with multifocal erosion (no ulcer)	25-50% of nasal epithelium is attenuated or necrotic. Focal ulceration possible.	>50% of nasal epithelium is necrotic, and/or evidence of multifocal ulceration
**Periodontal disease**	No or minimal periodontal and gingival inflammation	Inflammation affecting 10-25% of the maxillary gingiva and/or teeth on section	Inflammation affecting 25-50% of the gingiva and/or teeth, and occasionally extends to the bone	Inflammation affecting >50% of the gingiva and/or teeth, and/or dental abscess, fistula formation. Osteomyelitis common.
	**Absent**		**Present**	
**Trauma**	No evidence of nasal trauma		Maxillary fracture and/or significant subcutaneous or intranasal hemorrhage	
**Septal deviation**	The septum runs straight along the midline		The septum is deviated >10º or interrupted	
**Intraluminal bacterial colonies**	Individual bacteria may be present, but clear colonies are not observed		The lumen contains prominent bacterial colonies measuring ≥50 µm in diameter	
**Foreign body**	No evidence of a foreign body		Foreign body with inflammation	
**Epithelial hyper- or metaplasia**	Hyperplasia or metaplasia is absent or focally affecting <10% of the respiratory epithelium		Hyperplasia or metaplasia is multifocally affecting ≥10% of the respiratory epithelium	
**Active bone resorption**	Absent or small numbers of osteoclasts (≤2 per 10 HPF - 1.96 mm^2). No Howship's lacunae or bone lysis		Moderate (3-4 per 10 HPF) to large (≥5 per 10 HPF - 1.96mm^2) numbers of osteoclasts. Howship's lacunae and/or bone lysis	
**Vomeronasal organ (VNO) involvement**	VNO is within normal limits.		VNO showing inflammation and/or necrosis	

[a]Rhinitis cases were further classified according to inflammation type (i.e. neutrophilic, neutrophilic to mixed, fibronecrotizing, …) and whether inflammation was uni- or bilateral.
Abbreviation: HPF, high power fields

Supplemental Table S2. Antibiogram results from nasal bacterial isolates obtained from European hedgehogs (*Erinaceus europaeus*) with clinical respiratory disease.

Bacterial isolate	URTD	Bacterial Growth	Amoxi-clav	Sulfa-trim	Enrofloxacin	Marbofloxacin	Oxytetracycline	Gentamycin	Clindamycin	Cefalexin
Aeromonas encheleia	NO	Moderate Pure	RES	SUS	INT	INT	SUS	SUS	NI	RES
E. coli	YES	Heavy Mixed	RES	RES	INT	SUS	RES	SUS	RES	SUS
E. coli	NO	Light Pure	RES	SUS	SUS	SUS	RES	SUS	RES	SUS
E. coli	YES	Heavy Mixed	INT	SUS	SUS	SUS	SUS	SUS	RES	SUS
E. coli	YES	Heavy Mixed	INT	SUS	SUS	SUS	SUS	SUS	RES	SUS
E. coli	YES	Heavy Mixed	RES	SUS	SUS	SUS	SUS	SUS	RES	SUS
E. coli	YES	Heavy Mixed	RES	RES	SUS	SUS	SUS	SUS	RES	RES
E. coli (BH)[a]	YES	Heavy Pure	RES	RES	INT	INT	SUS	SUS	RES	RES
E. coli (BH)[a]	YES	Moderate Pure	RES	RES	INT	INT	SUS	SUS	RES	RES
E. coli	NO	Heavy Mixed	RES	RES	RES	RES	RES	SUS	RES	SUS
Mammalicoccus sciuri	NO	Heavy Mixed	RES	SUS	INT	SUS	SUS	SUS	SUS	SUS
Mammalicoccus sciuri	YES	Heavy Mixed	RES	SUS	INT	SUS	SUS	SUS	INT	SUS
Mammalicoccus sciuri	NO	Moderate Mixed	SUS	SUS	SUS	SUS	SUS	SUS	SUS	SUS
Mammalicoccus sciuri	YES	Heavy Mixed	RES	SUS	SUS	SUS	INT	SUS	INT	RES
Mammalicoccus sciuri	NO	Heavy Mixed	SUS	SUS	INT	INT	SUS	SUS	INT	SUS
Morganella morganii[a]	YES	Moderate Pure	RES	SUS	SUS	SUS	RES	SUS	RES	RES
Morganella morganii	YES	Moderate Mixed	RES	SUS	SUS	SUS	SUS	SUS	RES	RES
Pasteurella canis	YES	Heavy Mixed	SUS	SUS	SUS	SUS	SUS	SUS	RES	SUS
Pasteurella canis	YES	Moderate Mixed	SUS	SUS	SUS	SUS	SUS	SUS	RES	SUS
Pasteurella multocida[a]	YES	Heavy Pure	SUS	SUS	SUS	SUS	SUS	SUS	RES	SUS
Pasteurella multocida	YES	Moderate Mixed	SUS	SUS	SUS	SUS	SUS	INT	RES	SUS
Pasteurella multocida	YES	Moderate Mixed	SUS	SUS	SUS	SUS	INT	SUS	RES	SUS
Salmonella enteritidis	YES	Moderate Mixed	SUS	SUS	SUS	SUS	SUS	INT	RES	INT
Staphylococcus aureus	NO	Moderate Mixed	RES	SUS	SUS	INT	SUS	SUS	SUS	RES
Staphylococcus xylosus	YES	Moderate Mixed	SUS	SUS	RES	RES	SUS	SUS	INT	SUS

Abbreviations: URTD, animals diagnosed with histologically significant upper respiratory disease as described in the manuscript; Amoxi-clav, amoxicillin-clavulanic acid; Sulfa-trim, sulfamethoxazole-trimethoprim; SUS, susceptible; INT, intermediate; RES, resistant; NI, not interpretable; BH, beta-hemolytic

[a] Cases which had URTD combined with moderate to heavy, pure bacterial growth, suggesting direct association between the bacterial isolate and corresponding nasal lesions.
